# The varicella vaccination pattern among children under 5 years old in selected areas in china

**DOI:** 10.18632/oncotarget.17317

**Published:** 2017-04-21

**Authors:** Chenyan Yue, Yan Li, Yamin Wang, Yan Liu, Linsheng Cao, Xu Zhu, Kathryn Martin, Huaqing Wang, Zhijie An

**Affiliations:** ^1^ The Department of National Immunization Programme, Chinese Center for Disease Control and Prevention, Beijing, 100050, China; ^2^ Hangzhou Center for Disease Control and Prevention, Hangzhou, Zhejiang, 310021, China; ^3^ China Office of United Nations Children's Fund, Beijing, 100600, China

**Keywords:** varicella vaccine, vaccination, coverage, children, GDP

## Abstract

**Background:**

Vaccine is the most effective way to protect susceptible children from varicella. Few published literature or reports on varicella vaccination of Chinese children exist. Thus, in order to obtain specific information on varicella vaccination of this population, we conducted this survey.

**Methodology:**

We first used purposive sampling methods to select 6 provinces 10 counties from eastern, middle and western parts of China with high quality of Immunization Information Management System (IIMS), and then randomly select children from population in the IIMS, then we checked vaccination certificate on-site.

**Principal Findings:**

Based on the varicella vaccination information collected from 481 children's vaccination certificates from all ten selected counties in China, overall coverage of the first dose of varicella vaccine was 73.6%. There is a positive linear correlation between per capita GDP and vaccine coverage at county level (r=0.929, P < 0.01). The cumulative vaccine coverage among children at 1 year, 2 years and ≥3 years old were 67.6%, 71.9% and 73.6% respectively (*X*^2^=4.53, *P* =0.10). The age of vaccination was mainly concentrated in 12-17 months.

**Conclusions:**

The coverage rate of the first dose of varicella vaccine in selected areas was lower than that recommended by WHO position paper. The coverage rate was relatively low in areas of low social-economic status. The cumulative coverage had no significant statistical difference among different age group. Most children received varicella vaccine before 3 years old. We suggest introducing the varicella vaccine into routine immunization program, to ensure universal high coverage among children in China. We also suggest that varicella vaccination information should be checked before entering school, in order to control and prevent varicella outbreaks in schools.

## INTRODUCTION

Varicella is an infectious disease with high transmissibility, common in children [1]. The disease is prone to resulting in large scale outbreak in institutional units, such as nurseries, kindergartens, and schools [2]. Varicella vaccine has been demonstrated good safety and effect [3], and varicella vaccination is the most effective measure to protect susceptible people from the disease. Although varicella case information has been collected nationally since 2005 through National Disease Supervision Information Management System(NDSIMS), its limitation in quality of local clinic reporting poses a challenge in accurate analysis and evaluation of the true situation in China [4]. Based on the estimation of varicella incidence in Shandong, Gansu and Hunan provinces, 4,705,000 cases were reported in 2007 in China, and the cost estimation in 2007 was 2.31 billion RMB for outpatients and 103 million RMB for inpatients [5, 6]. “Varicella and Herpes Zoster Vaccines: WHO Position Paper” recommends that countries where varicella is an important public health burden could consider introducing varicella vaccination in the routine childhood immunization programme. Resources should be sufficient to ensure reaching and sustaining vaccine coverage ≥ 80%. Vaccine coverage that remains <80% over the long term is expected to shift varicella infection to older ages in some settings, which may result in an increase of morbidity and mortality despite reduction in total number of cases [3]. Varicella vaccine was licensed in China in 1996 [7]. Currently, varicella vaccine is sold on the private market, meaning it is voluntary and must be self-paid. There is no national recommended immunization schedule available for varicella vaccine, and the vaccine is administered to eligible children at a vaccination clinic according to instructions provided by the vaccine manufacturers. Based on the immunization regulation, the record of vaccination information is kept by both the parents and the clinic [8–9]. In this study, we investigated the varicella vaccination information among children under 5 years old in selected areas in China, in order to understand the varicella vaccination situation for children in China. This information will provide evidence for authority department of China introducing varicella vaccine into the national routine immunization program.

## RESULTS

### Basic information and vaccine coverage in selected counties

**Figure 1:**
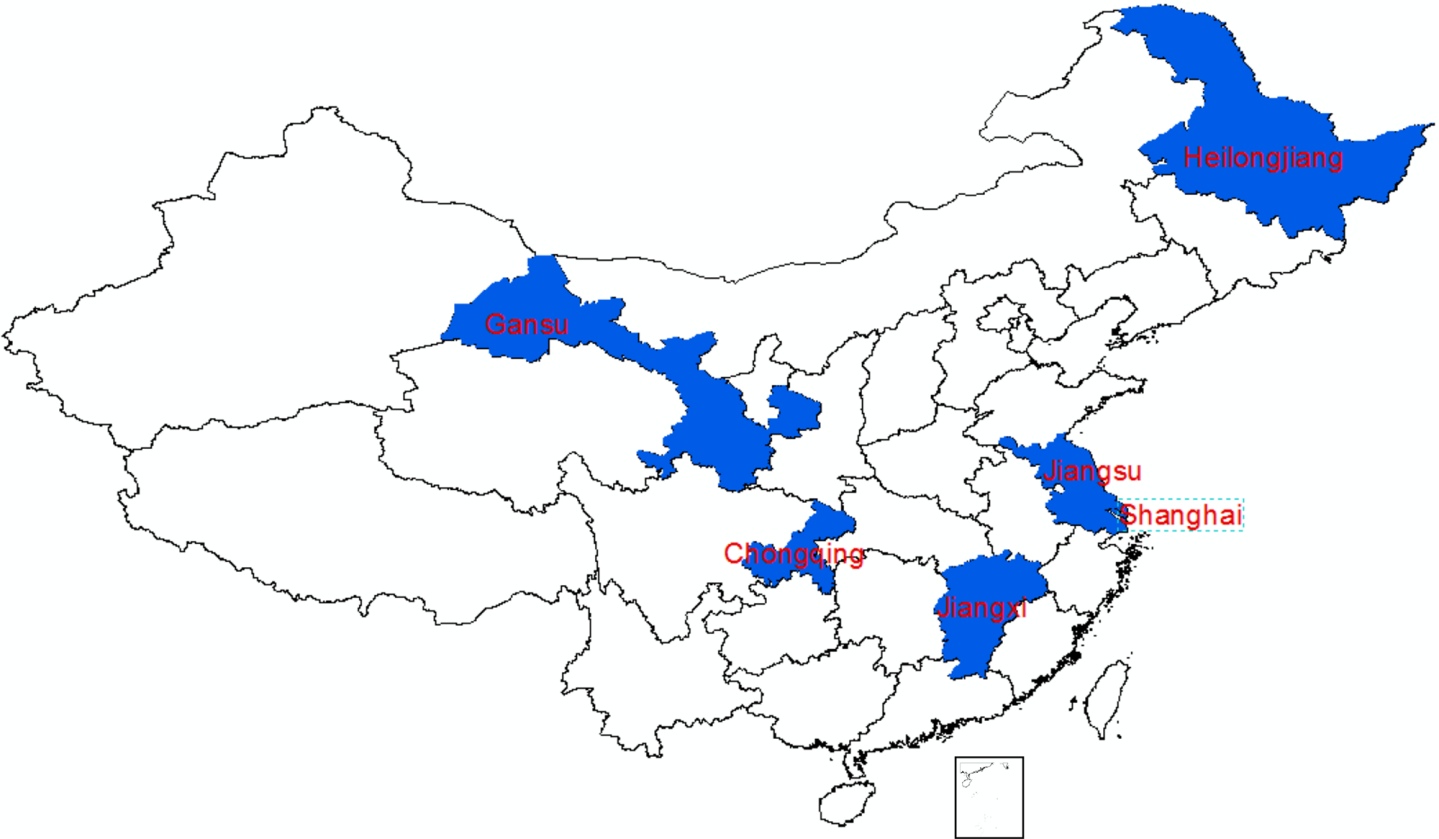
The geographical distribution of the selected provinces in China

In total, we collected varicella vaccination information from 481 caregivers’ vaccination certificates: 96 in Shanghai, 48 in Jiangsu, 96 in Heilongjiang, 52 in Jiangxi, 93 in Gansu and 96 in Chongqing, the geographical distribution of selected provinces see Figure 1; Male: 263 and female: 218, with a male to female ratio of 1.2:1. The varicella vaccine coverage of the first dose was 73.6% (354/481) for all children surveyed. The coverage in selected counties is described in Table 1. 1.2% (6/481) received the second dose of varicella vaccine.

**Table 1:** Coverage among children born between 2008 and 2012 in the selected counties

Province	County (district)	No. of children investigated	No. of children vaccinated	Coverage Rate (%)
Shanghai	Huangpu	48	46	95.8
	Changning	48	43	89.6
Jiangsu	Jianye	48	41	85.4
Heilongjiang	Ning'an	49	26	53.1
	Suifenhe	47	46	97.9
Jiangxi	Donghu	52	30	57.7
Gansu	Chengguan	43	19	44.2
	Qilihe	50	27	54.0
Chongqing	Nan'an	47	38	80.9
	Shapingba	49	38	77.6
Total		481	354	73.6

### Relationship between varicella vaccine coverage and the economic situation

**Figure 2:**
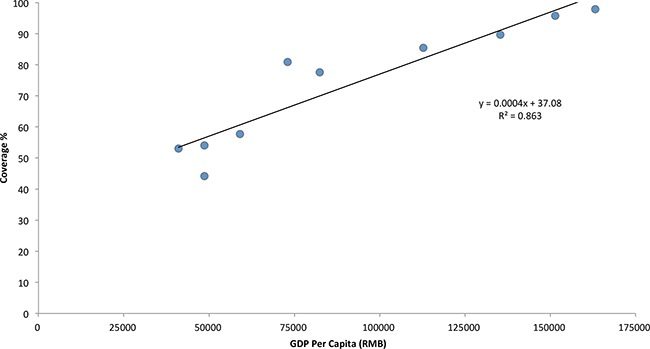
The relationship between varicella vaccine coverage and the economic status in the selected areas

The median coverage of varicella vaccine in areas with per capita GDP ≥ 100,000 RMB is 92.7% (85.4% - 97.9%), while median coverage of varicella vaccine in areas with per capita GDP < 100,000RMB is 55.9% (44.2% - 80.9%) (Wilcoxon rank sum test P < 0.05). There is a positive linear correlation between per capita GDP and vaccine coverage at county level (r = 0.929, P < 0.01). The equation of linear regression is y = 0.0004x + 37.08 (F = 50.426, P < 0.001) (Figure 2).

### Cumulative coverage of varicella vaccine and distribution of vaccinated age

At age of 1 year old(12-23 months), the vaccine coverage were 70.8%, 70.0%, 65.7%, 70.0%, 60.5% among children born in 2008, 2009, 2010, 2011 and 2012 respectively with no significant difference (*X *^2^ = 3.15, *P* =0.53). The cumulative vaccine coverage ≥3 years old (≥ 36 months) were 79.2%, 78.0% and 74.7 % among children born in 2008, 2009 and 2010 respectively with no significant difference (*X *^2^=0.59, *P* =0.75). The children born in 2012 were under 2 years of age during investigation for whom the vaccine coverage was lower (60.5%). In total, the cumulative coverage among children at 1 year, 2 years(24-35 months) and≥3 years old were 67.6%, 71.9% and 73.6% (*X *^2^=4.53, *P* =0.10), with 4.3% increasing at 2 years old, and 1.7% at 3 years old and later compared to 1 year old (Table 2).

**Table 2:** Cumulative coverage of varicella vaccine among children born between 2008 and 2012 in the selected areas

Birth date	No. of children investigated	No. of children vaccinated				Cumulative coverage (%)		
		1 year* old	2 years* old	≥3 years* old	Total	1 year* old	2 years *old	≥3 years* old
2008	96	68	5	3	76	70.8	76.0	79.2
2009	100	70	6	2	78	70.0	76.0	78.0
2010	99	65	6	3	74	65.7	71.7	74.7
2011	100	70	4	-	74	70.0	74.0	-
2012	86	52	-	-	52	60.5	-	-
In total	481	325	21	8	354	67.6	71.9	73.6

Note:* 1 year old=”12-23 months”, 2 years old=”24-35 months”, ≥3 years old=”≥36 months”

**Figure 3:**
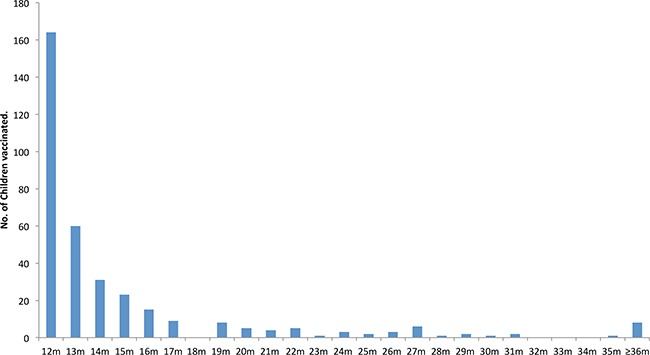
The distribution by vaccinated age (months) for children who received varicella vaccine in the selected areas n=(ta/2Δp)2P(1−P),ta/2=1.96,Δp=0.0045,p=0.45

Among all vaccinated children, 91.8% (325/354) received the vaccine at 1 year old, 5.9% (21/354) received at 2 years old, and 2.3% (8/354) at 3 years old and later. Among those receiving the vaccine at 1 year old, the vaccinated age was mainly concentrated in 12-17 months, accounting for 85.3% (302/325) (Figure 3).

## DISCUSSION

The basic reproduction number (R_0_) is about 8 ∼ 10 for varicella, which is higher than influenza [10], it is easy for varicella to cause an outbreak in a nursery, kindergarten or school. Therefore, it is very difficult to block the spreading of varicella disease in areas with low vaccine coverage, and it is necessary to maintain high coverage to prevent the disease from becoming an epidemic. The World Health Organization position paper states that varicella may transit to older age groups when the rate of vaccination coverage is lower than 80% for a significant amount of time, which may result in increased morbidity and mortality [3]. During this survey we found that the coverage of the varicella vaccine was higher in counties with higher per capita GDP, and was relatively lower in counties with lower per capita GDP. The coverage of selected areas varies from 44% to 98%, and basing on the linear regression modeling of the relationship between GDP and coverage, per capita GDP of China was 38,354 RMB in 2012 [11], we estimated the average coverage rate for children under 5 years old in China might be about 52%. A national survey conducted in 2011 in China showed that the varicella vaccine coverage rate under 2 years old was 47% [12], this is similarly with our estimates.

Varicella vaccine is a private vaccine in China, the parents of children voluntarily select and pay for the vaccine for their children. In areas with low coverage rate, a child will be individually protected due to the vaccination, however, herd immunity may not be established at the population level. Furthermore, as a private vaccine, the vaccination fee may become a barrier to vaccinate for children who live in financially poor resource environments [13, 14]. The inequalities of immunization coverage due to socioeconomic differences also exist in other countries [15, 16]. Varicella vaccine has been demonstrated good safety and effect [3], and one study in China showed that introducing varicella vaccination into the routine childhood immunization programme is quite cost effective: an one-dose strategy could save 8 billion RMB in one year [17]. We suggest introducing the varicella vaccine into the routine childhood immunization program as soon as possible to improve the equity in access to immunization services in areas of low socioeconomic status, and ensure a coverage rate of more than 80% in all areas according to the recommendations by WHO.

Our study showed that the coverage rate for different birth cohorts remained stable. The vaccine coverage rate was 68% at 1 year old, and increased slightly with age, cumulative vaccine coverage rate was 72% at 2 years old and 74% ≥ 3 years old, suggesting that after 3 years of age the access to varicella vaccination service is limited. The peak seasons for incidence of varicella in China are winter and spring seasons, and mainly effect preschool and school-age children of 3 ∼ 10 years old [18]. In China, varicella outbreaks account for a relatively high proportion of the school emergency public health events. For example, reported outbreaks due to varicella in Zhejiang province accounted for 35% in the school emergency public health events from 2005 to 2008 [19]. Therefore, it is necessary to provide an opportunity for access to varicella vaccination service for those children over 3 years of age, who missed the opportunity for vaccination at a younger age. School entry check of immunization certificates and providing vaccination services for eligible children during entry of kindergarten and school may increase vaccine coverage rate among older children and reduce the amount of outbreaks in nurseries and schools [20].

At present, the one-dose schedule for varicella vaccine is used in most provinces in China, and the one-dose schedule is used in the 6 provinces selected for this investigation. This survey found that most children completed the schedule before 2 years of age, with the majority (85%) receiving the vaccine at 12-17 months old. Research in the United States showed that the vaccine efficacy decreased significantly (only 73%) during the first year after vaccination if the vaccine is provided before 15 months old [10]. Before 2006, the one-dose schedule was recommended in the United States. After 2006, in order to strengthen the control of varicella, a two-dose schedule was introduced, the first dose is administered at 12-15 months old and the second dose administered at preschool age (4-6 years old) [21]. Geometric mean titer (GMT) showed a particularly high boost after the second dose when the interval between doses was more than one year [22]. A two-dose schedule was introduced in Beijing in 2012: the first dose at 18 months of age and the second dose at 4 years of age [7]. To further improve and optimize the varicella vaccine schedule, we suggest further developing the recommendations regarding the varicella immunization schedule by considering disease risk and the effectiveness of vaccines.

Limitations: We used purposive sampling methods to select provinces and counties (districts) [23], the selected provinces cannot represent all of China. And we obtained the sample based on Immunization Information Management System (IIMS), according to the national requirements, all immunization information for children should be reported to IIMS, however, the quality of the IIMS data varies across areas, it is possible that some migrant children's information was not recorded in IIMS, which may overestimate the coverage rate of varicella vaccine.

## MATERIALS AND METHODS

### Sampling methods

We used purposive sampling methods to select counties (districts) from Shanghai, Jiangsu, Heilongjiang, Jiangxi, Chongqing and Gansu provinces. We selected these investigation sites based on two factors: (i) representation of east, middle and western parts of China; (ii) having good records of private vaccinations in children's vaccination certificates and with a high quality Immunization Information Management System (IIMS). From Nov 1^th^ 2013 to Nov 15^th^ 2013, we used the simple random sampling method to randomly select children from the population whose birth date was between Jan 1^st^ 2008 and Dec 31^th^ 2012 in the IIMS in selected counties (districts), then from January 1^th^ 2014 to July 15^th^2014, we checked hand-held vaccination certificates on-site to collect varicella vaccination information.

We used the following formula to calculate the sample size:

$$\begin{array}{c} n={\left(\frac{t_{a/2}}{\Delta_{p}}\right)}^{2}P(1-P),\\ t_{a/2=1.96,}\Delta_{p=0.0045,}p=0.45 \end{array}$$

n: the sample size, P: estimate coverage of varicella vaccine, based on past experience, we used 45%. After calculation, a sample size of 470 was needed for this survey.

Considering a loss to follow-up of about 10%, a total of 520 children needed to be investigated. The sample size was divided by 10 (10 counties), and 52 children in each county were selected to participate in the investigation. Finally, 39 children were excluded because their parents did not have a hand-held vaccination certificate or we were unable to contact them.

### Calculation of per capita gross domestic product (GDP)

Per capita GDP=GDP/domicile population. GDP and household population data came from the “2012 Per Capita National Economic and Social Development Statistical Bulletin” on the selected County or District's Bureau of Statistics website. The data was not available for Jianye in Jiangsu, Ning'an in Heilongjiang, Chengguan and Qilihe in Gansu, so the per capita GDP in the corresponding city level (higher administrative level) was used for these counties.

### Survey content and data processing

We conducted this survey to obtain the basic information of children (such as gender, date of birth) and varicella vaccination (such as inoculation date and doses). We used Excel to enter the data, ArcGIS to draw the map, and Epi Info software to analyze the data.

### Ethics statement

We obtained oral informed consent from all children's guardians before the investigation, and two investigators checked vaccination certificates on-site together. We would continue the investigation only when the children's guardians gave consent. We didn't go through the institutional review board approval in the case of this study for ethical issues, because monitoring vaccine coverage is part of routine program work, in this context, there is no risk for participants and it is not required to get IRB approval. However, we strictly protected the private information of participants in the field, such as the name and contact information. During the analysis, we also took out the personal identification information.
